# Hypothyroidism is a Risk Factor for Atrial Fibrillation after Coronary Artery Bypass Graft

**DOI:** 10.21470/1678-9741-2017-0080

**Published:** 2017

**Authors:** Marisol Carreno Jaimes, Luis Alberto Arciniegas Torrado, Néstor Fernando Sandoval Reyes, Jaime Camacho Mackenzie, Juan Pablo Umana Mallarino

**Affiliations:** 1 Fundación Cardioinfantil - Instituto de Cardiología, Bogota, Colombia.

**Keywords:** Coronary Artery Bypass, Atrial Fibrillation, Hormones, Hypothyroidism

## Abstract

**Introduction:**

Few reports in the world have shown a differential effect of hypothyroidism in relation to morbidity and mortality following cardiac surgery.

**Objective:**

To determine the association between preoperative hypothyroidism, composite and disaggregated outcomes of mortality and complications in patients undergoing first-time isolated myocardial revascularization surgery.

**Methods:**

Historical cohort of patients undergoing myocardial revascularization between January 2008 and December 2014, with 626 patients included for evaluation of the composite and disaggregated outcomes of in-hospital mortality and complications (atrial fibrillation, surgical site infection and reoperation due to bleeding). A logistic regression model was used to determine the association between hypothyroidism and the onset of those outcomes.

**Results:**

Cohort of 1696 eligible patients for the study, with 1.8 mortality. Median age, female gender and prevalence of arterial hypertension were all significantly higher among hypothyroid patients. No differences were found in other preoperative or intraoperative characteristics. Hypothyroidism was associated with the presence of the composite outcome, RR 1.6 (1.04-2.4) and atrial fibrillation 1.9 (1.05-3.8). No association with mortality, infections or reoperation due to bleeding was found.

**Conclusion:**

Hypothyroidism is a disease that affects females predominantly and does not determine the presence of other comorbidities. Hypothyroidism is a risk factor for the onset of postoperative fibrillation in patients undergoing myocardial revascularization surgery. Postoperative care protocols focused on the prevention of these complications in this type of patients must be instituted.

**Table:** 

Abbreviations, acronyms & symbols	
CABG	= Coronary artery bypass surgery
CPB	= Cardiopulmonary bypass circulation
STS	= Society of Thoracic Surgeons
TSH	= Thyroid stimulating hormone
WHO	= World Health Organization

## INTRODUCTION

According to the World Health Organization (WHO), cardiovascular disease accounts for 46% of deaths from noncommunicable diseases in the world, which means that 7.4 million people die of a coronary event^[1]^.

In Colombia, according to the National Health Institute, 56.3% of deaths in the cardiovascular disease group were due to coronary heart disease, this figure being higher that the cumulative figure reported by the WHO^[2]^.

However, the biggest concern for governments and healthcare services relates to the millions of people who survive a coronary event and who are in need of specific treatment for the disease, secondary prevention, and rehabilitation^[1]^.

Multiple factors have been described, including genetics, the environment, life style and even geography as influencing coronary artery disease^[1]^. The disease consists of atheroma plaques that line the length and bifurcations of the proximal and distal branches of coronary circulation. This creates an imbalance in oxygen supply which results in a process of ischemia, injury and, finally, infarction and death of the myocardial fibres when sufficient oxygen supply cannot be restored^[3]^.

One of the interventions for treating coronary heart disease is coronary artery bypass surgery (CABG)^[4]^. This operation has been shown to produce excellent results in terms of mortality, with a 10-year survival of 80%, making it a safe procedure^[5]^.

Although this operation - performed with growing frequency due to the logarithmic increase in the number of cases of coronary heart disease - has been shown to be safe, the frequency of complications may be as high as 13%, meaning that 13 out of 100 patients undergoing this procedure may experience at least one complication postoperatively^[4]^.

Multiple patient-related and/or procedure-related factors have been reported in the literature as affecting the onset of complications and mortality during the postoperative period^[4,5]^.

Moreover, differences have been shown between patients with and without metabolic diseases in terms of mortality and complications following CABG, with an association between the presence of those diseases and a higher frequency of complications^[6]^.

One of the patient-related factors is the presence of hypothyroidism before CABG. Although the association between the presence of coronary heart disease and thyroid dysfunction is well known, it is not clear yet whether that dysfunction may have an impact postoperatively^[6]^.

Although figures on the hypothyroid population are not well known, there are reports showing a higher frequency of coronary heart disease in patients with metabolic diseases such as diabetes mellitus and hypothyroidism^[6,7]^.

No studies has yet been published in Colombia or Latin America about the impact of hypothyroidism on surgical procedures. There are only a few reports in other cardiac surgery centres in the world showing the same differential impact of hypothyroidism in terms of mortality and morbidity following cardiac surgery^[6-10]^.

The objective of this study was to determine the association between a preoperative diagnosis of hypothyroidism and the presence of the composite and disaggregated outcome of mortality and complications (atrial fibrillation, surgical site infection, reoperation due to bleeding) in patients undergoing first-time myocardial revascularization.

## METHODS

### Study Design and Population

Retrospective cohort study of patients undergoing firsttime isolated CABG. Exposed patients were those who met at least one of the following criteria: a) a history of pre-operative hypothyroidism, b) a diagnosis made during the preoperative clinical history, c) a quantitative elevation of the thyroid stimulating hormone (TSH) found up to seven days before the surgical procedure. Non-exposed patients were those who did not meet any of the three criteria listed above. The end-points of the study were: composite and disaggregated outcome of inhospital mortality and complications (atrial fibrillation, surgical site infection and reoperation due to bleeding).

Eligibility criteria: Exclusion criteria were patients taken to emergent surgery; patients who had died in the operating room or within the first 24 hours after surgery; patients with hybrid surgery and/or minimally invasive surgery.

### Sample Size and Sampling

Sample size was estimated using the Sample Size software version 1.1 and using the arcsine method for estimating two proportions for cohort studies with the following parameters: Type I error, 0.05; Type II error, 0.2; group-control proportion, 0.12; rate of assignment between the groups, 1:1; type of estimation, 2 tails; parameter for the exposed group, relative risk 2; total number of patients in the exposed group, 314; total number of patients in the non-exposed group, 314. The systematic nonprobabilistic sampling of all the patients undergoing CABG at the cardiovascular surgery service between January 2008 and December 2014 was designed to identify exposed patients. A random sampling was used for non-exposed patients, using the order of the date of surgery as the systematic criterion during the same study period.

### Statistical Analysis

A data audit was conducted in order to look for outliers, missing values, mismatches or error data. The audit was based on a random selection of 10% of the total number of patients included, and each of the variables was verified against the clinical record. The overall audit error was 0.7%, resulting in the approval of the information.

A univariate exploratory data analysis was conducted as follows: after using the Shapiro-Wilk test to verify distribution type, quantitative variables were expressed as the mean plus standard deviation for variables with normal distribution, and as median plus inter-quartile range for variables without a normal distribution. Qualitative variables were expressed in absolute and relative frequencies. A comparative analysis of the exposed group (hypothyroid patients) *versus* the non-exposed group (non-hypothyroid patients) was conducted in order to look for differences among preoperative, intraoperative and postoperative variables.

Quantitative variables with normal distribution were compared using Student's T test, and variables with a non-parametric distribution were compared using the Mann - Whitney test. Qualitative variables were compared using the Chi square test.

A bivariate analysis was performed to determine individual variable association with the composite and disaggregated outcome of in-hospital deaths and complications (atrial fibrillation, surgical site infection and reoperation due to bleeding). Independent variables were entered in the logistic regression model when the *P* value was lower than 0.2 at that point of the analysis. A logistic regression model was applied to determine the association between hypothyroidism and the outcomes, adjusted for preoperative and intraoperative variables.

An evaluation of the confounding variables of sex, age and use of cardiopulmonary bypass circulation (CPB) was performed. No significant or clinically relevant interactions were observed. The stepwise strategy was used, starting with the complete model. Regression diagnoses were performed and the quality of the model was evaluated once each variable was eliminated, using the R^2^, Akaike and Bayesian criteria, and ending with the Hosmer and Lemeshow test.

The results of the associations were expressed in relative risks with their respective 95% confidence intervals. The statistical tests were considered significant, with a *P* value of less than 0.05. The analyses were carried out using the STAT 13.0 software package.

### Ethical Considerations

The study was conducted in accordance with the principles set forth in the 18^th^ World Medical Assembly (Helsinki, 1964) and its subsequent amendments, and with Resolution 8430 of 1993 of the Colombian Ministry of Health. According to Article 11 of the latter regulation, this research was considered free of risk. Respect for the dignity of the individuals and protection of the patient's privacy and confidentiality rights prevailed throughout the different phases of the study.

The study protocol was reviewed and approved by the Research Committee and by the Ethics Committee of Fundacion Cardioinfantil - Instituto de Cardiología. The research was conducted by professionals in training under the supervision of competent professionals with knowledge and experience to ensure the privacy and quality of patient information. The information was used purely for academic purposes and the confidentiality will be preserved.

## RESULTS

**Fig. 1:**
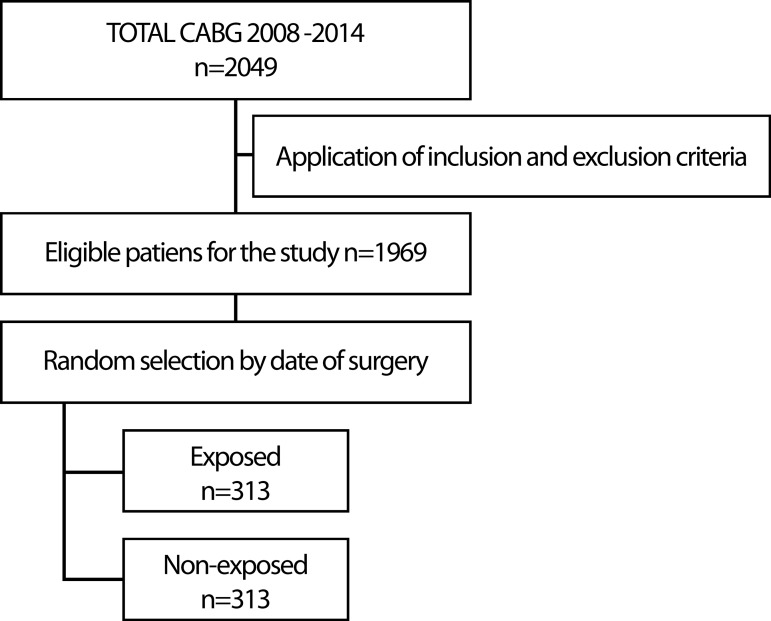
Study sampling flowchart.

During the study period, 2049 first-time isolated myocardial revascularization procedures were performed at Fundación Cardioinfantil - Instituto de Cardiología. Inclusion and exclusion criteria were applied for the selection of the patients of the exposed group (hypothyroid) and the non-exposed group (non-hypothyroid). Figure 1 shows the sampling flow-chart, and the patients who were included in the analysis.

The overall prevalence of hypothyroidism in the general cohort from which the study patients were selected was 15.9% (n=313). The general characteristics of the population are described below, stratified according to the presence of hypothyroidism, as shown in Table 1.

**Table 1:** Preoperative characteristics of the participants.

Variable	Total N= 626	Non-hypothyroid N= 313	Hypothyroid N= 313	*P* Value
Age in years. Median (IQR)	65 (58-72)	63 (56-70)	68 (61-73)	0.0001
Female sex n (%)	168 (26.8)	57 (18.2)	111 (35.5)	0.0001
Arterial hypertension, Yes n (%)	474 (75.8)	219 (69.9)	255 (81.5)	0.001
Diabetes Mellitus, Yes n (%)	237 (37.9)	108 (34.5)	129 (41.2)	0.08
Body Mass Index kg/m^2^ Median (IQR)	25.5 (23.7-27.9)	25.7 (23.5-27.7)	25.4 (23.7-28.0)	0.68
Dyslipidemia, Yes n (%)	350 (55.9)	165 (52.7)	185 (59.1)	0.107
Heart Failure, Yes n (%)	27 (4.3)	10 (3.2)	17 (5.4)	0.17
Myocardial infarction, Yes n (%)	349 (55.7)	170 (54.3)	179 (57.2)	0.47
Creatinine, mg/dl. Median (IQR)	0.92 (0.8-1.1)	0.9 (0.8-1.1)	0.92 (0.8-1.2)	0.77

N=number; IQR=inter-quartile range

The analysis of the general population revealed a cohort of young patients with a high proportion of hypertension, diabetes and dyslipidemia as common factors in the pathophysiology of cardiovascular disease, as well as a high proportion of myocardial infarction as the presenting symptom.

In the hypothyroid group, the mean age of the patients was higher, with a greater proportion of women and hypertensive patients than in the non-exposed group, and these differences were statistically significant. Regarding the other variables, although there was a trend towards increased frequency among hypothyroid patients, the difference was not statistically significant. Intraoperative characteristics were similar in the two groups, as shown in Table 2.

**Table 2:** Variables related to the surgical procedure.

Variable	Total N= 626	Non-hypothyroid N= 313	Hypothyroid N= 313	*P* Value
Use of cardiopulmonary bypass, Yes n (%)	402 (64.2)	190 (60.7)	212 (67.7)	0.07
Aortic clamping time (Minutes). Median (IQR)	71 (57-87)	70 (56-84)	72 (58-88)	0.06
CPB time (Minutes). Median (IQR)	90 (75-108)	89 (76-108)	91 (76-109)	0.15
Use of intra-aortic balloon pump counterpulsation, Yes n (%)	19 (2.0)	10 (3.2)	9 (2.9)	0.82

N=number; IQR=Inter-quartile range; CPB=cardiopulmonary bypass

Statistically significant differences in the proportion of the composite outcome and the proportion of the atrial fibrillation outcome could be noted, which were higher in the group of hypothyroid patients, as shown in Table 3.

**Table 3:** Postoperative outcomes.

Variable	Total N=626	Non-hypothyroid N=313	Hypothyroid N= 313	*P* Value
Composite outcome*, Yes. n (%)	119 (19.0)	47 (15.0)	72 (23)	0.01
In-hospital mortality, Yes. n (%)	12 (1.9)	4 (1.3)	8 (2.6)	0.24
Postoperative atrial fibrillation, Yes. n (%)	83 (13.3)	29 (9.3)	54 (17.2)	0.003
Surgical site infection, Yes. n (%)	30 (4.8)	11 (3.5)	19 (6.1)	0.13
Reoperations due to bleeding, Yes. n (%)	22 (3.5)	8 (2.6)	14 (4.5)	0.19

N=Number.

* Death, atrial fibrillation, surgical site infection, reoperation due to bleeding.

Mortality in the original cohort of patients was 1.8% (37/1969), and in-hospital mortality and surgical site infection, although more frequent in hypothyroid patients, did not show a statistically significant difference.

Table 4 describes the final model that confirms hypothyroidism as a risk factor for the presence of the composite outcome and postoperative atrial fibrillation adjusted for covariables. The variables explain the outcomes by more than 80% according to the R2 analysis, and the result of the proof of adequacy of the adjustment hypothesis was not significant, suggesting that the model adjusts adequately.

**Table 4:** Association of hypothyroidism with the composite outcome and atrial fibrillation. Co-variable-adjusted model.

Variable	Composite outcome		Atrial fibrillation	
	OR (95% CI)	*P* value	OR (95% CI)	***P*** value
Hypothyroidism, Yes	1.6 (1.04-2.4)	0.03	1.9 (1.05-3.8)	0.04
Age¥, years	1.7 (1.04-2.7)	0.03	2.0 (1.02-4.0)	0.04
CPB time, Min*			1 (1.19-6)	0.02
R2	81%		84%	
AIC	0.96		0.86	
BIC	-3414		-1674.5	
Adjustment goodness	0.80		0.66	

CPB=cardiopulmonary bypass; Min=minutes; AIC=Akaike information criterion; BIC=Bayesian information criterion

¥ Over 61 years of age.

* CPB longer than 123 minutes, adjusted for CPB use.

## DISCUSSION

Aging of the world population and the associated burden of disease create an opportunity to generate new knowledge designed to comprehend and attempt to reduce the burden of the disease, in this case of chronic non-communicable diseases.

The assessment and understanding of the comorbidities of the aging population affected by cardiovascular events are the basis for planning primary and secondary prevention strategies, improving patient care and rehabilitation, and ensuring that medical, interventional and/or surgical treatments yield the maximum expected benefits^[11]^.

This study evaluated the impact of preoperative hypothyroidism on the outcomes of CABG in terms of mortality and morbidity. The objective was to determine whether the presence of this metabolic comorbidity increased the occurrence of death and complications, as has been observed in patients with metabolic syndrome and diabetes^[12-14]^.

The patients in the study were similar to those reported in the American registries of the Society of Thoracic Surgeons (STS) and in European, Asian and Japanese studies, with similar ages and prevalence of comorbidities such as hypertension, diabetes and dyslipidemia^[15-18]^.

The overall prevalence of hypothyroidism in this study was higher than that reported by Zindrou et al.^[7]^ and Park et al.^[8]^ at a range between 0.5-11%, adjusted by sex. This may be explained on the basis of the preoperative assessment protocols, because the use of a systematic assessment may improve detection and diagnosis of patients with hypothyroidism, as is the case in our own institution.

Hypothyroid patients were older than the controls and there were more women with this diagnosis. These findings are similar to those reported by Zindrou et al.^[7]^, Park et al.^[8]^, and Ning et al.^[19]^, and they may be associated with the fact that this metabolic disease is more frequent in females and, moreover, the onset of coronary heart disease tends to occur later in women due to the protective effect of oestrogens^[7,8,19]^.

No important differences was found in terms of other comorbidities between hypothyroid and non-hypothyroid patients. As suggested by Zindrou et al.^[7]^ and Park et al.^[8]^, this metabolic disease does not determine the onset of other diseases, unlike what has been observed in diabetes and heart failure^[12,13,20]^.

No differences was found either in terms of the surgical characteristics between the groups, showing that the technical conditions of the procedure were not impacted by the presence of this diagnosis in this cohort of patients. The results of the operative characteristics are comparable to those reported in American series^[11,15]^.

The mortality observed in the original cohort of the patients selected for study, and the mortality in hypothyroid patients is similar to the mortality reported in the STS and the European registries^[15,16]^.

Although mortality in hypothyroid patients is higher than in non-exposed patients, it is not significantly different from that observed by Park et al.^[8]^. However, it is in contrast to what has been observed for other cardiovascular procedures^[20-22]^.

The incidence of complications such as atrial fibrillation, reoperation due to bleeding and surgical site infection in the cohort is similar to what has been reported in American and European series^[15,16]^. There was a difference between the groups only for the postoperative atrial fibrillation outcome, but this finding has not been observed in prior studies^[20,22]^.

This study showed a risk association between hypothyroidism and the onset of atrial fibrillation as comorbidity-adjusted independent factor. Comorbidities affecting the association between hypothyroidism and the presence of the composite outcome and atrial fibrillation were age over 61 years for both outcomes, and bypass circulation time greater than 123 minutes for atrial fibrillation.

The strengths of this study are the fact that it was conducted in an institution with national and international quality accreditation, with an average volume of 400 cardiac surgeries per year, by a surgical team that has remained stable during this time period; an institutional prospective and systematic collection database for all the patients in the study; and similar mortality outcomes as those reported in the literature. All of these factors result in less variability of care, safety of the procedures, and quality of the information^[23,24]^.

The limitations of the study, derived from its retrospective nature, were controlled by the selection of a cohort coming from a prospective data collection with the same operationalization protocol for the variables of all the patients; outcome stratification in accordance with clinical and subclinical hypothyroidism which requires an *ad hoc* study that will be conducted once the sample size is completed for the stratified outcomes; the assessment of other factors that could impact the magnitude of the association of hypothyroidism; and the assessment of other outcomes.

## CONCLUSION

Hypothyroid patients taken to myocardial revascularization surgery are older and have a higher prevalence of arterial hypertension than the non-hypothyroid population.

Hypothyroidism is a disease that affects females predominantly but is not a determining factor for the presence of other comorbidities apart from the ones mentioned above.

Mortality in patients undergoing myocardial revascularization surgery in this institution is comparable to that observed in American and European series.

Hypothyroidism is an independent, comorbidity-adjusted risk factor for the onset of postoperative atrial fibrillation in patients undergoing myocardial revascularization.

In this study, hypothyroidism was not a factor associated with the occurrence of mortality, surgical site infection or reoperation due to bleeding.

Further studies are needed to evaluate the long-term impact of this comorbidity in patients undergoing myocardial revascularization in order to determine whether it affects the survival of the procedure.

**Table:** 

Authors' roles & responsibilities	
MCJ	Substantial contributions to the conception or design of the work; or the acquisition, analysis, or interpretation of data for the work; final approval of the version to be published
LAAT	Substantial contributions to the conception or design of the work; or the acquisition, analysis, or interpretation of data for the work; final approval of the version to be published
NFSR	Substantial contributions to the conception or design of the work; or the acquisition, analysis, or interpretation of data for the work; final approval of the version to be published
JCM	Substantial contributions to the conception or design of the work; or the acquisition, analysis, or interpretation of data for the work; final approval of the version to be published
JPUM	Substantial contributions to the conception or design of the work; or the acquisition, analysis, or interpretation of data for the work; final approval of the version to be published
